# Endothelial Dysfunction in Severe Preeclampsia is Mediated by Soluble Factors, Rather than Extracellular Vesicles

**DOI:** 10.1038/s41598-017-06178-z

**Published:** 2017-07-19

**Authors:** Michelle O’Brien, Dora Baczyk, John C. Kingdom

**Affiliations:** 1 The Research Centre for Women’s and Infant’s Health, Lunenfeld Tanenbaum Research Institute, Mount Sinai Hospital, University of Toronto, Toronto, Canada; 2 The Department of Obstetrics & Gynecology, Maternal-Fetal Medicine Division, Mount Sinai Hospital, University of Toronto, Toronto, Canada

## Abstract

In severe early-onset preeclampsia (sPE) the placenta releases soluble angiogenesis-regulating proteins, trophoblast-derived fragments, and extracellular vesicles (EVs). Their relative importance in disease pathogenesis is not presently understood. We explanted placental villi from healthy and sPE women then separated the media into: total-conditioned, EV-depleted and EV-enriched media. Three fractions were compared for; angiogenic protein secretion by ELISA, angiogenic and inflammation gene mRNA expression and leukocyte adhesion assay. sPE placental villi secreted significantly less PlGF (70 ± 18 pg/mL) than preterm controls (338 ± 203; p = 0.03). sFlt-1:PlGF ratios in total-conditioned (115 ± 29) and EV-depleted media (136 ± 40) from sPE placental villi were significantly higher than in EV-enriched media (42 ± 12; p < 0.01) or any preterm or term media. Fluorescent-labeled EVs derived across normal gestation, but not from sPE, actively entered HUVECs. From sPE placental villi, the soluble fraction, but not EV-enriched fraction, significantly repressed angiogenesis (0.83 ± 0.05 fold, p = 0.02), induced *HO-1 mRNA* (15.3 ± 5.1 fold, p < 0.05) and induced leukocyte adhesion (2.2 ± 0.4 fold, p = 0.04). Soluble media (total-conditioned and EV-depleted media) from sPE placental villi induced endothelial dysfunction in HUVEC, while the corresponding EV-enriched fraction showed no such effects. Our data suggest that soluble factors including angiogenesis-regulating proteins, dominate the vascular pathology of this disease.

## Introduction

Severe early-onset preeclampsia (PE) is a rare hypertensive disorder of pregnancy characterized by systemic endothelial dysfunction that results in temporary ischemic injury of many body organ systems and is distinct from the milder and more common form of the disease presenting at term^1^. Considerable advances have been made in the past decade to understand the mechanisms by which the diseased placenta causes life-threatening severe hypertension in PE^2^. In the classic disease characterized by histologic features of maternal vascular malperfusion in the placenta^3, 4^, the villous trophoblast layer covering the placental villi forms aggregates of syncytial knots that release excessive amounts of the anti-angiogenic soluble fms-like kinase-1 (sFlt-1) into the maternal circulation^5–7^. In tandem, the placental release of pro-angiogenic placental growth factor (PlGF) is impaired^8, 9^.

An alternate pathway by which the placenta may mediate abnormal systemic vascular function is via the release of particulate structures from the syncytiotrophoblast surface. The human placenta is capable of releasing a range of syncytial debris into maternal blood^10–12^, including large multi-nucleated aggregates (20–500 µm)^13^, that are mostly filtered in the lung^14^. A wide range of smaller micro-vesicles, across the size range 40–2,000 nm, are also released by the human placenta into maternal blood. Originally described as syncytiotrophoblast microparticles (STBM)^15, 16^, by virtue of their size these structures will cross the lung capillary bed to enter the systemic vasculature which may impact cellular function at distal sites. A subset of smaller particles, which includes cell surface-derived micro-vesicles (50–1000 nm) and the actively secreted nano-particles of endosomal origin (40–120 nm) called exosomes^17^, are collectively referred to as extracellular vesicles (EVs). In addition to size differences, individual vesicle types vary in cellular origin, composition and biological function^17, 18^.

The potential for STBM fractions, passing through the maternal lungs, to interact with the maternal endothelium in an endocrine manner, has been under active investigation since the early 1990s. An early widely-cited report demonstrated that STBMs isolated from blood of healthy control and PE women equally suppressed endothelial cell proliferation and disrupted the cultured endothelial cell monolayer^15^. Some investigators have reported elevated amounts of STBMs in maternal blood of PE women^16, 19, 20^, whereas others report no differences between normal and hypertensive pregnancies^21, 22^. Numerous investigators attempted to link the circulating STBMs to the pathogenesis of PE^16, 22–24^. When subjected to endothelial cells *in-vitro*, concentrated amounts of STBMs isolated from maternal blood or perfused term placentas, exert deleterious effects (for detailed review see –ref. 11). Additionally, PE derived trophoblast debris was shown able to activate endothelial cells^25^.

EVs, in particular, are of increasing interest as a mechanism of cell-cell communication, since they are capable of transferring proteins, lipids and nucleic acids into the recipient cells thereby influencing their physiological and pathological function^18, 26^. The role of circulating EVs in mediating physiological adaptive mechanisms in normal pregnancy (elegantly summarized in refs 13 and 26) is of considerable interest, for example in mediating maternal immune tolerance to the semi-allogenic developing fetus. EVs play an important role in breast cancer progression^27^, likely via local paracrine signaling mechanisms that support both tumor proliferation and host tissue angiogenesis^28^.

*In vitro* preparations of EVs are heterogeneous by nature, comprising of both surface-derived micro-vesicles and actively secreted exosomes. Isolation of these smaller particles is often plagued by the contamination of other cell products, such as high density lipoproteins and protein aggregates, which accumulate in the preparations resulting from ultracentrifugation isolation methods^29^. These contaminants may confound results and therefore must be removed from the EV preparations. Recent advancements in the standardized isolation, purification and characterization methods have facilitated a more uniform approach to obtaining fractions that are enriched for exosomes and micro-particles. In particular, studies have revealed that ultracentrifugation followed by size-exclusion chromatography yield the most pure EV preparations compared to ultracentrifugation alone and precipitation methods^30, 31^.

Thus far, studies have been unable to distinguish the potential effects of soluble proteins from the effects of STBMs and/or EVs. Such biologically distinct fractions, secreted by placental villi into maternal blood, require evaluation at their original secretion rates in order to compare their potency as endocrine mediators of maternal systemic endothelial cell injury. Here we have prepared soluble and EV fractions directly from placental villi to define their relative importance in mediating the pathogenesis of the underlying vascular dysfunction and hypertension observed in pregnancies complicated by severe early-onset preeclampsia.

## Results

### Isolation and characterization of EVs derived from floating human placental villi

**Figure 1:**
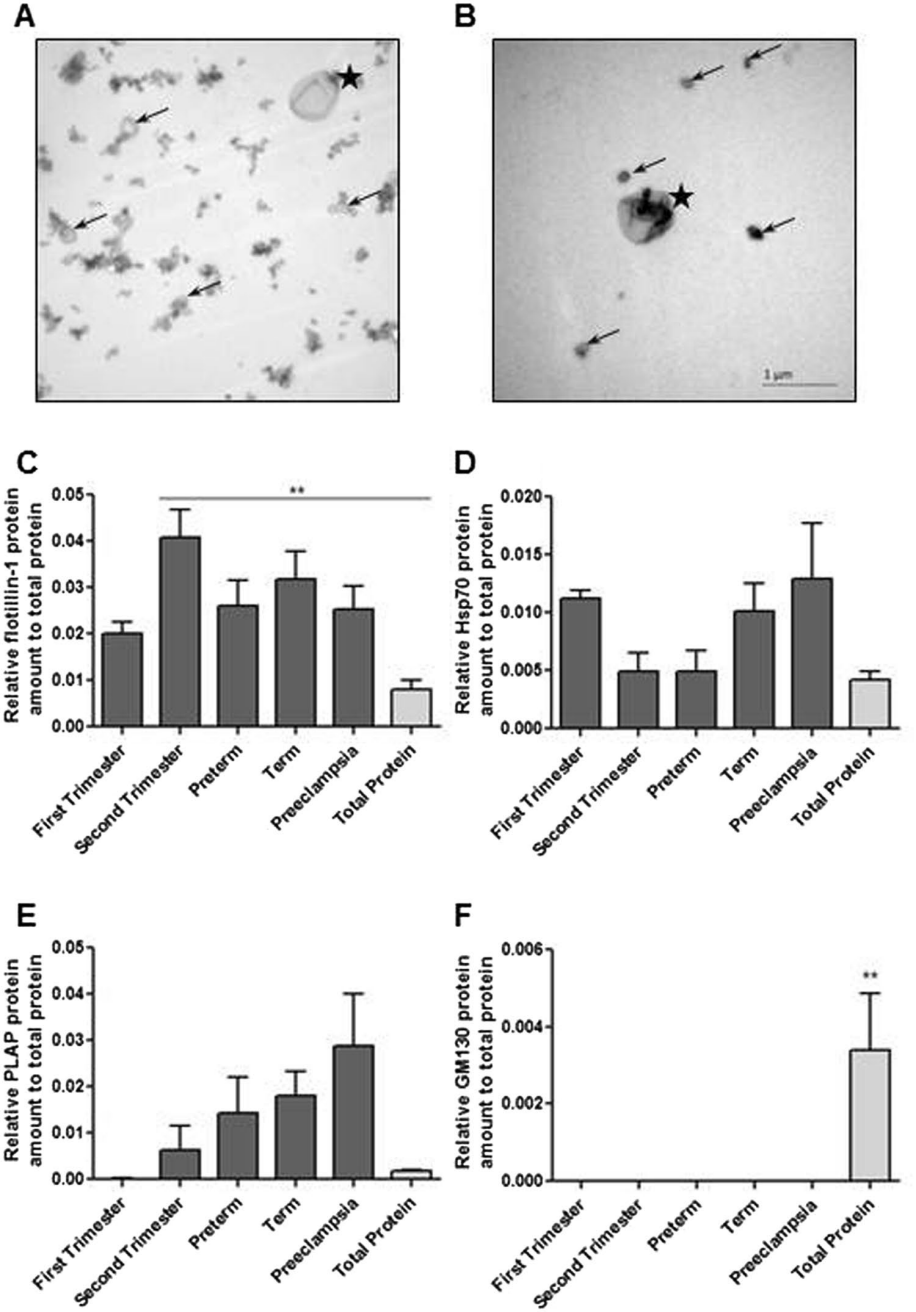
Purity of EVs was assessed using low magnification TEM. (**A**) EV preparation prior to and (**B**) following qEV exclusion column. Images taken at 19,000X magnification. Arrows = exosome, * = micro-vesicles. (**C**–**F**) Additional characterization of the EVs was performed by Western blotting for EV-specific markers, Flotilin-1 and Hsp70, and for placenta specific marker-PLAP. Purity of preparations was further validated with GM130 antibody. Densitometry analysis for (**C**) Flotilin-1, (**D**) Hsp70, (**E**) PLAP and (**F**) GM130 are presented.

EV fraction was successfully isolated from conditioned media collected from explanted floating human placental villi utilizing a series of spins and purifications steps (Supplementary Figure 1). Transmission electron microscopy visualized EVs (Fig. 1A) and demonstrated the importance of the qEV purification step to remove protein aggregates (Fig. 1B). Western blot analyses (Supplementary Figure 2) for EV-specific proteins Flotillin-1 and Hsp70 (Fig. 1C and D) respectively, demonstrated elevated levels of EV markers in all of the EV preparations compared to total tissue protein lysate (pooled data across all gestational ages and sPE placental explants). No specific patterns in expression were observed in any group (Fig. 1C–E). The Golgi marker GM130 (Fig. 1F), used to monitor for contaminants, was not expressed in any of the EV-enriched fractions. From these experiments we conclude that EVs, derived from floating human placental villi, can be successfully extracted and purified for further analysis across gestation and in sPE.

### Fluorescently-labeled trophoblast-derived EVs across normal human gestation, but not from severe preeclamptic pregnancies, actively enter HUVEC

**Figure 2:**
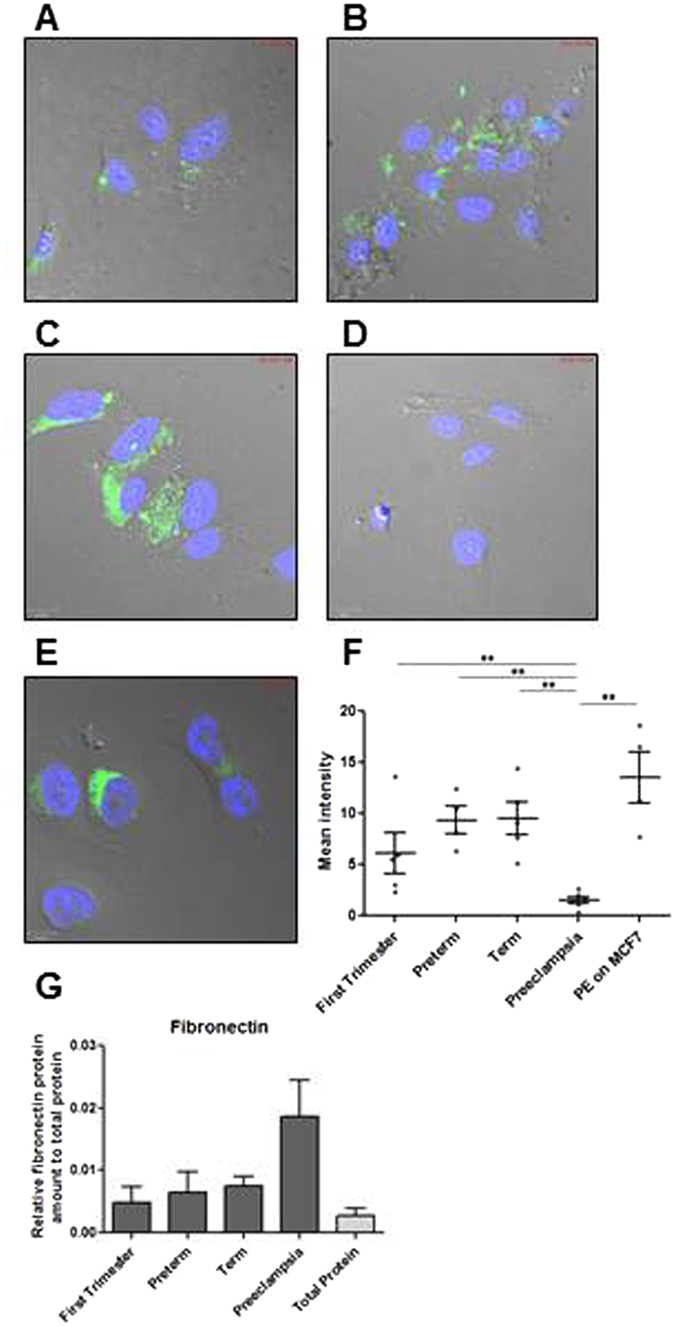
PKH67 fluorescently labelled EVs generated from (**A**) first, (**B**) preterm, (**C**) term and (**D**) PE placental explants were incubated with HUVEC cells for 4 hrs. (**E**) Pre-eclamptic EVs were also incubated with MCF7 breast cancer cell line for 4 hrs. (**F**) PE-derived EVs are unable to enter HUVEC cells but are able to enter into cancer cells. (**G**) EVs generated from PE express fibronectin.

In order to test the biologic viability of our purified EVs, we monitored the entry of fluorescently labelled EVs into target endothelial cells. EVs purified from placental villi of normal pregnancies across gestation successfully entered target HUVEC cells (Fig. 2A–C). By contrast to healthy term EVs (mean intensity 9.5 ± 1.6 n = 5), EVs derived from sPE placental villi showed minimal entry into HUVEC (Fig. 2D; 1.5 ± 0.3 n = 6, p < 0.01) yet successfully entered a control breast cancer cell line MCF7 (13.5 ± 2.5 n = 4, p < 0.19) (Fig. 2E, quantified in 2F). The same experiments were successfully reproduced using a uterine microvascular endothelial cell line and T47D breast cancer cell line (data not shown).

Since fibronectin mediates EV uptake in recipient cells^32^ we hypothesized that deficient entry of EVs derived from sPE placental villi was due to lack of fibronectin expression. However, analysis of fibronectin protein expression levels in EV preparation from healthy and PE placentas revealed similar level of fibronectin in PE EVs as compared to preterm and term controls (PE 0.019 ± 0.006 n = 5, preterm 0.006 ± 0.03 n = 3, term 0.008 ± 0.002 n = 7) (Fig. 2G), suggesting that deficient fibronectin cannot explain the inability of PE EVs to enter endothelial cells.

### Angiogenic protein expression in total-conditioned, EV-depleted and EV-enriched media

**Figure 3:**
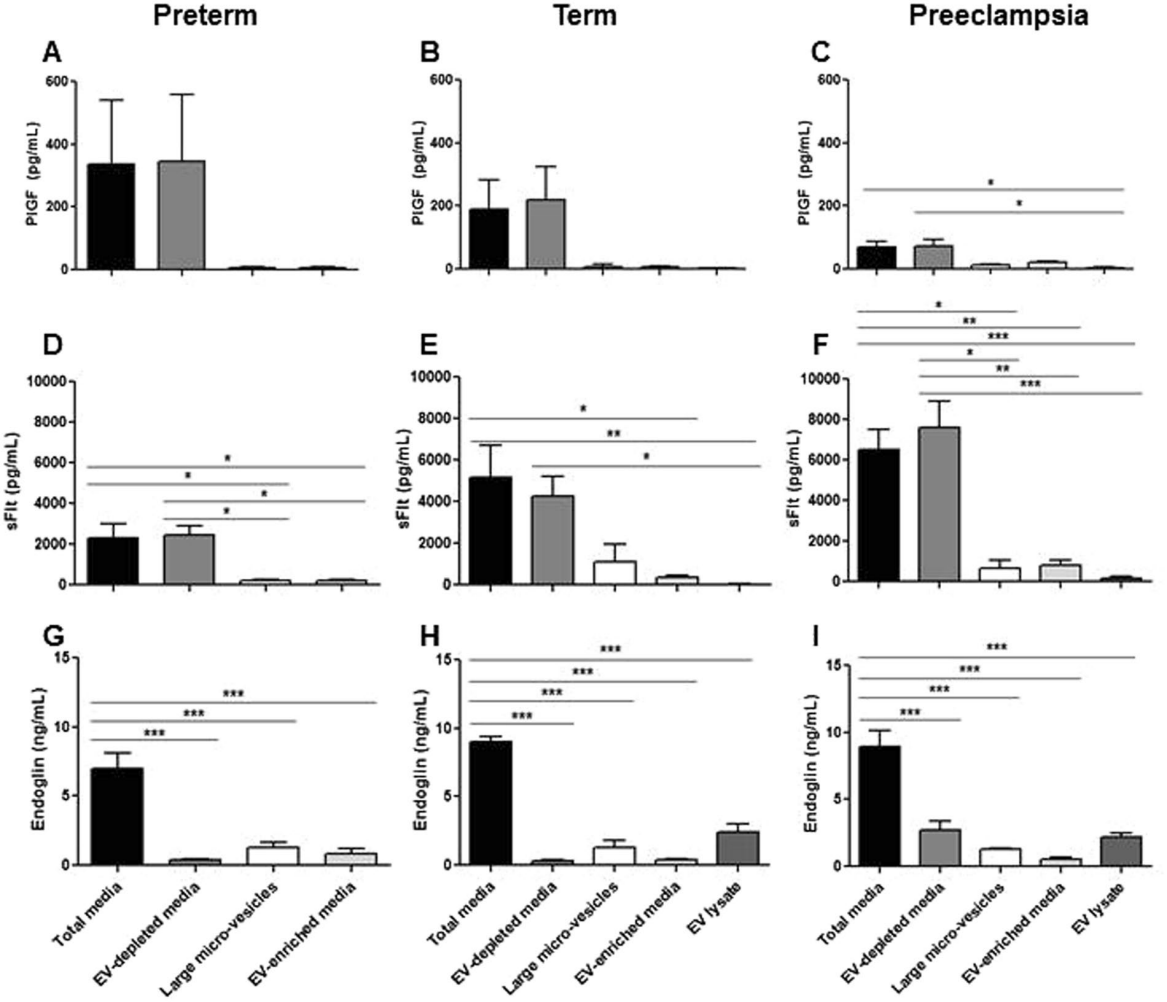
Media analysis by ELISA for (**A**–**C**) PlGF, (**D**–**F**) sFlt-1 and (**G**–**I**) Endoglin from preterm, term and PE placenta. PlGF and sFlt-1 levels were mainly detected in total and EV-free media. Endoglin was detected in total media but its levels were markedly reduced in EV-depleted media. All three angiogenic proteins showed minimal levels in EV-enriched media, microvesicle-enriched media and EV lysates. Large micro-vesicles = obtained after the 10,000 g spin, include micro-vesicle and apoptotic bodies. EV lysates = obtained by RIPA lysis of purified EV pellets.

**Figure 4:**
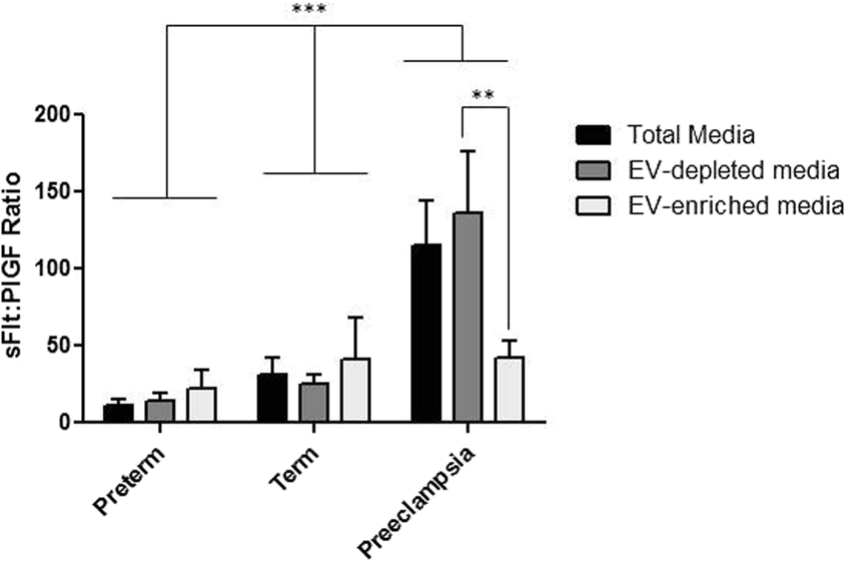
sFLt-1:PlGF ratio in media preparations from preterm, term and sPE placentas. NS = not significant.

**Figure 5:**
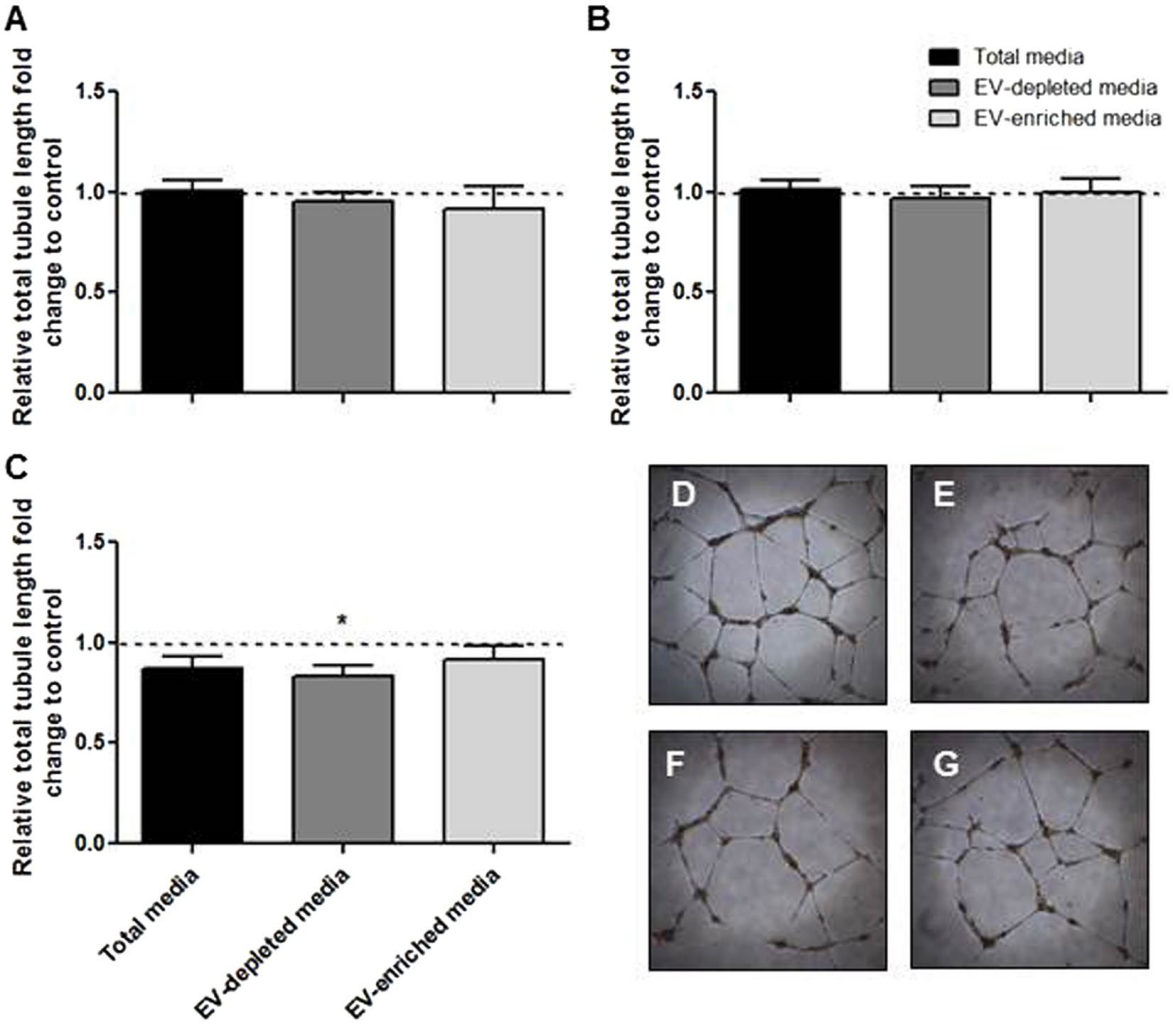
Angiogenic activity of HUVECs treated with (**A**) preterm or (**B**) term conditioned and enriched media was not altered. (**C**) HUVEC angiogenesis was moderately decreased when treated with PE conditioned media but not EV-enriched media. Representative images of (**D**) positive control, (**E**) total PE media, (**F**) EV-depleted media and (**G**) PE-EV enriched media.

Total media analysis by ELISA demonstrated a highly significant reduction in PlGF secretion by sPE placental villi compared to preterm or term conditioned media (sPE = 70 ± 18 pg/mL, preterm = 338 ± 203 pg/mL, term = 188 ± 94 pg/mL, p = 0.03, n = 4) (Fig. 3A–C). PlGF is soluble as it was almost undetectable in ELISA preparations from both large micro-vesicles and EV-enriched media. Media analysis of sFlt-1 secretion showed a significant increase in sPE media compared to preterm controls (Fig. 3D–F). The majority of secreted sFlt-1 is soluble, with a small fraction in both large micro-vesicles and EV enriched media. In total-conditioned media, the sFlt-1:PlGF ratio from sPE placental villi was significantly elevated compared with preterm placental villi (Fig. 4). sFlt-1:PlGF ratios remained significantly elevated in EV-depleted media from sPE placentas but was significantly reduced in EV-enriched media, to values similar to media from preterm and term placental villi, in the normal range established for maternal blood in the third trimester^33^. Endoglin production in total-conditioned media was similar in sPE and normal placental villous tissues (Fig. 3G–I). Endoglin was markedly absent in EV-depleted media, mostly expressed in micro-particle fractions. Media conditioned by preterm and term (Fig. 5A and B respectively) placentas did not negatively affect angiogenesis determined by total tube length formation. Within sPE media, significant angiogenesis repression was confined only to EV-depleted media (0.83 ± 0.05 fold, n = 7, p = 0.02) (Fig. 5C and F). 10-fold concentrated EV-enriched preparations from sPE placental villi did not affect HUVEC angiogenesis (data not shown).

### EV-depleted media, but not EV-enriched media, alters inflammatory gene expression and leukocyte adhesion

Prior to performing functional assays with the total-conditioned media, EV-enriched and EV-depleted media, the effect of the three preparations on the proliferation and toxicity in HUVECs was assessed (Supplementary Figure 3). Placenta conditioned media derived from healthy first trimester pregnancies exert a small significant increase in HUVEC proliferation as compared to vehicle control (1.18 ± 0.52 fold, n = 5, p = 0.03) (Supplementary Figure 3A), while placenta-conditioned media from severe PE pregnancies exert a small decrease in HUVEC proliferation although this was not statistically significant (0.91 ± 0.04 fold, n = 4, p = 0.12) (Supplementary Figure 3I); no effects on HUVEC cell toxicity were found (Supplementary Figure 3B,D,F,H,J).

**Figure 6:**
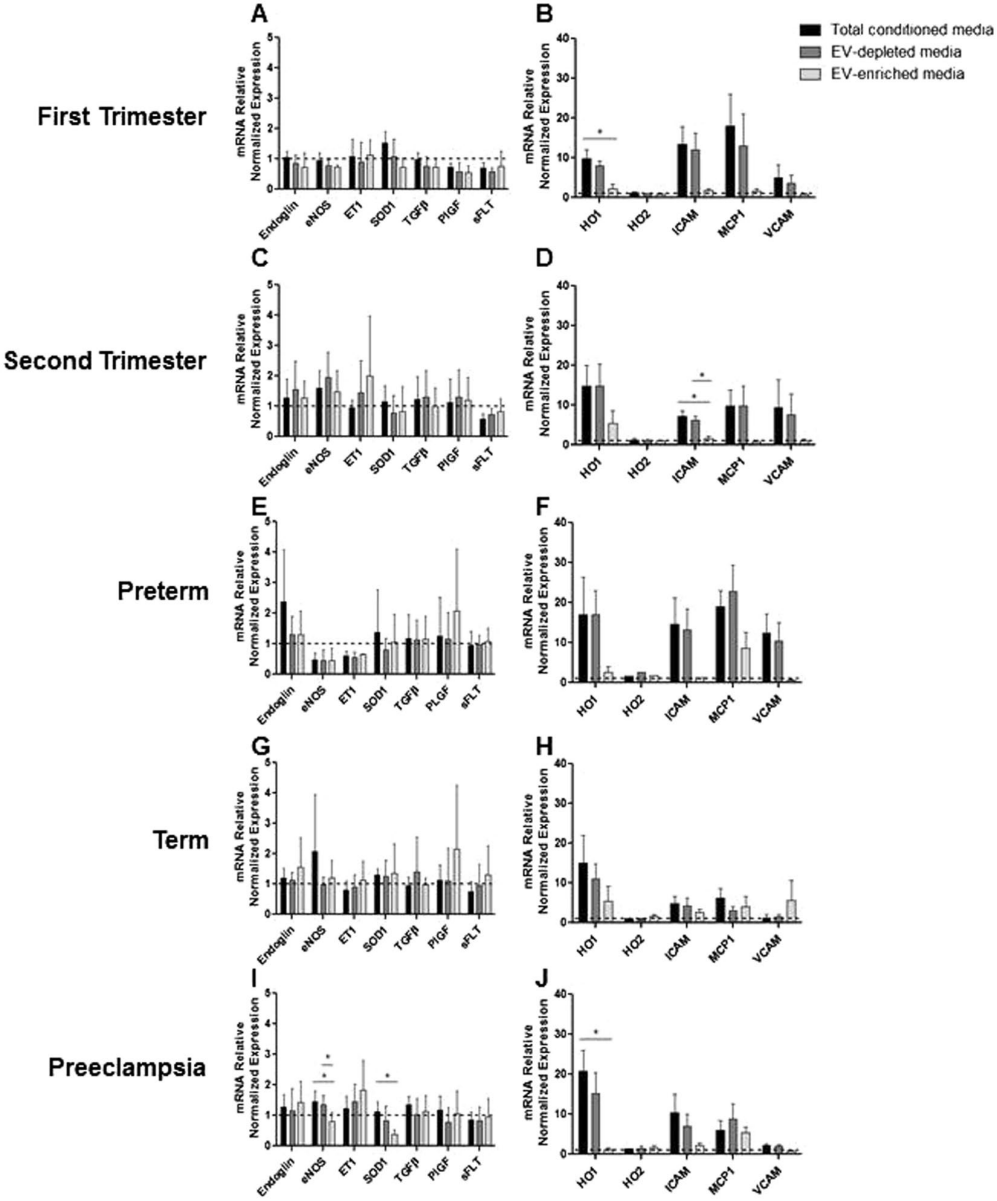
HUVECs were treated with total, EV-depleted and EV-enriched media from (**A**,**B**) first trimester, (**C**,**D**) second trimester, (**E**,**F**) preterm, (**G**,**H**) term and (**I**,**J**) PE placentas for 24 hr.

Minimal differences, likely of no biologic significance, were found in mRNA expression levels for a range of genes regulating endothelial cell function (*Endoglin*, *eNOS*, *Endothelin*, *SOD1*, *TFGß*, *PlGF and sFlt-1*) (Fig. 6, left column). By contrast mRNA expression of the inducible *HO1* mRNA (but not the non-inducible *HO2* mRNA) was found in total conditioned media and EV-depleted media from sPE explants (*HO1* total media: 20.9 ± 5.1 fold, EV-depleted: 15.3 ± 5.1 fold (n = 5, p < 0.05), vs EV-enriched media (1.3 ± 0.4, n = 5, NS) (Fig. 6J). Similar HO1 findings were found in first trimester media (Fig. 6B).

**Figure 7:**
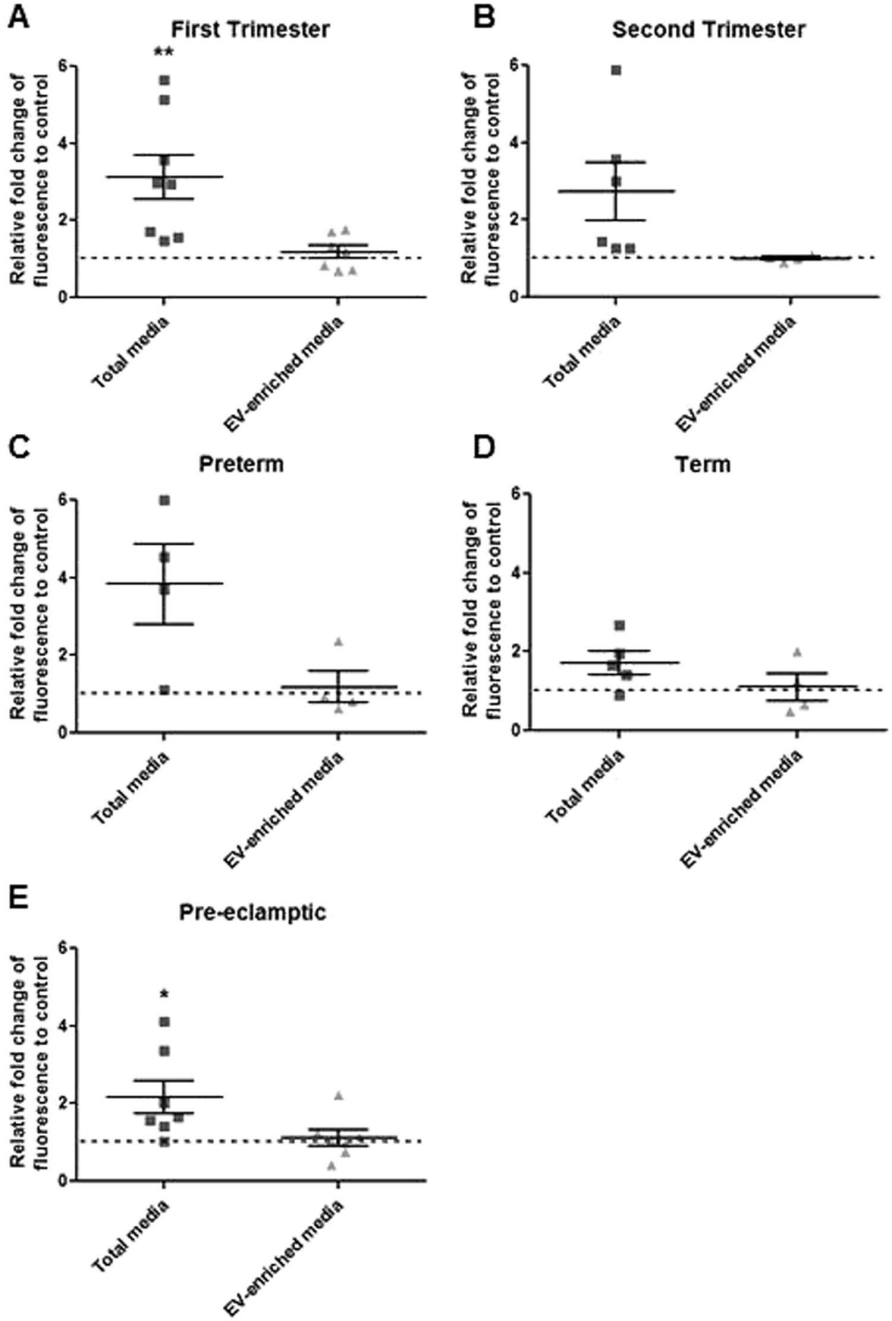
Endothelial cells were treated with total and EV-enriched media from (**A**) first trimester, (**B**) second trimester, (**C**) preterm, (**D**) term and (**E**) PE placentas for 6 hrs prior to incubation with fluorescently labelled monocytes for 45 min. First trimester and PE total media but not the EV-enriched media elicited leukocyte activation.

Total-conditioned media, but not EV-enriched media, demonstrated significant increase in leukocyte adhesion assay compared to vehicle control when prepared from both first trimester and sPE placental villi (first trimester: total media = 3.1 ± 0.6 fold, p = 0.007, n = 8, EV-enriched media = 1.2 ± 0.2 fold, p = 0.32, n = 7; sPE: total media = 2.2 ± 0.4 fold, n = 7, p = 0.04, EV-enriched media = 1.1 ± 0.2 fold, n = 7, p = 0.57) (Fig. 7).

## Discussion

Our study provides a standardized approach designed to distinguish the relative importance of placenta-derived soluble factors and EVs on endothelial cell function. Here, we have extended our previous work using the floating placental villous explant model^34^ to successfully separate the soluble fraction of conditioned media from the various classes of particles released by the syncytiotrophoblast layer that is in direct contact with maternal blood. In comparable media-conditioning concentrations, we demonstrate that soluble factors, rather than EVs, mediate *in-vitro* effects on the endothelium predicted to negatively affect endothelial cell function.

In support of the soluble factor basis of this disease, we demonstrate that placental villi from sPE have high secreted sFlt-1:PlGF ratios, very similar to levels found in circulating maternal blood *in-vivo* ^33, 35^. The reduction in sFlt-1:PlGF ratios into the normal range following preparation of the EV-enriched fraction, in contrast with preservation of abnormally elevated sFlt-1:PlGF ratios in EV-depleted media, indicates that our experimental approach separated soluble and EV fractions. These data support our conclusion that soluble factors from sPE placental villi exert negative effects on endothelial cells.

Our data do not exclude biologic roles for placenta-derived EVs in either normal or abnormal pregnancy, especially in paracrine signaling settings within the uterus, where local concentrations, perhaps derived from extracellular trophoblast within the uterine wall, are likely to be much higher than in circulating maternal blood. We were surprised that labeled EVs derived from sPE placentas failed to enter HUVEC cells. We did demonstrate that they entered a control breast cancer cell line and expressed fibronectin, however the mechanisms by which they fail to enter HUVEC is presently unknown. EVs could however be able to influence target cell function via surface interactions. EVs are capable of inducing IL-1ß in target macrophages via fibronectin-integrin surface receptors, without the need for internalization^36^. Furthermore, we could not demonstrate any substantial changes in angiogenesis gene mRNA expression in response to EV-enriched fraction. While not significant across all gestations; *ICAM*, *MCP1* and *VCAM* mRNA levels were elevated in total-conditioned media and EV-depleted media but not in the EV-enriched media. In support of our observations, Hoegh, *et al*.^37^ reported that healthy term placenta derived STBMs did not significantly influence HUVEC gene expression. Since total and EV-depleted but not EV-enriched media induced changes in *HO1* and some pro-inflammatory genes, we therefore conclude that the soluble fraction of conditioned media is most likely responsible for mediating impaired endothelial cell function in sPE.

Perhaps the placental hypoxia-ischemia injury that characterizes sPE^38^ alters the orderly production and release of EVs in sPE, including their protein and miRNA content (reviewed in ref. 11). Further studies are needed to explore this possibility. Rather than suggest that placental-derived EVs are biologically inert, we suggest that syncytiotrophoblast-derived EVs secreted into maternal blood could have important physiological functions in normal pregnancy, including participation in mediating maternal hemodynamic changes that protect against preeclampsia, in particular by reducing systemic vascular resistance^39^. In support of this hypothesis, EVs derived from the maternal circulation in normal pregnancy induced wound healing in a model with primary HUVEC cells^40^.

Our study has important limitations. First, our data interpretation is confined to HUVEC cells, which are derived from the human fetal umbilical circulation. We were able to recapitulate some aspects of our findings in human uterine microvascular endothelial cells, but we recognize that our findings will be greatly strengthened if they could eventually be applied to adult-derived endothelial cells from small resistance arteries. Another approach would be to study isolated perfused small arteries^41^, though at present a major limitation in performing these types of perfusion studies is the quantity of EVs that can be prepared from small villous explants.

Our data conflict with the conclusions of some investigators who have studied the potential role of STBMs in preeclampsia. We appreciate that maternal blood contains a complex mix of particles, including surface shed fragments and actively-secreted exosomes, from many cell types, including the immune system^42^. The complex interplay of all these signaling systems undoubtedly contributes to the pathogenesis of this heterogeneous disorder^43^. However, we believe that careful dissection of the potential role of individual organs, their cell types and produced/released vesicles is important in order to understand this disease, especially the placental villi, since they are strikingly-abnormal in sPE. Extensive research conducted by many investigators with either blood^15^ or placenta-specific derived STBMs^24, 44^ demonstrates the potential of the placental syncytiotrophoblast surface to damage endothelium. Our data advance our understanding of this disease as we have separated soluble and EV fractions from placental villi, the tissue mostly responsible for this disease. We encourage other investigators to adopt this approach so as to understand the potential for the various classes of placenta-derived micro-particles, including EVs, to mediate vascular damage via endocrine pathways after passage via the maternal lungs.

## Methods

### Placental Sample Collection

Healthy first and second trimester placental villous tissue samples were obtained from Morgentaler Clinic, Toronto, Canada, following voluntary legal termination of pregnancy. Gestational age and viability were established pre-operatively by ultrasound. Preterm, term and placental tissues from women with severe early-onset preeclampsia (sPE) were collected following deliveries at Mount Sinai Hospital, Toronto, Canada. Patient characteristics are summarized in Supplementary Table 1. Women with sPE met the ACOG definition of this disease^45^. All women gave written informed consent; methods were performed in accordance with the relevant guidelines and regulations as set out and approved by ethics board of Mount Sinai Hospital, Toronto, Canada (MSH 11-0248-E). Tissue specimens were obtained by the Research Centre for Womens’ and Infants’ Health BioBank program. Immediately following termination or delivery, placental villous tissues were dissected and briefly rinsed in chilled phosphate-buffered saline (PBS) to remove excess blood. All samples were processed within 4 hrs.

### Explant Culture and EV Isolation Protocol

Approximately 1000 mg of healthy and preeclamptic placental villous explants (3–4 explants, 10–15 mg per explant) were cultured in 12-well plates for 72 hours in 2.5 mL of serum-free placental explant media (DMEM HAM F12 supplemented with 1% gentamycin, 0.1% fungicide, 1% insulin/transferrin/selenium, 1% penicillin/streptomycin/L-glutamine; (Thermo Fisher, Burlington, ON, Canada)) at 8%O_2_ as previously reported^34^.

The isolation and characterization of EVs was performed using criteria established by the International Society of Extracellular Vesicles^31^. After 72 hours of culture, the media was spun at 300 g for 10 minutes at 4 °C to remove cells and larger cell debris, as outlined in Supplementary Figure 1. A sample of media collected at this point was defined as “total cultured media preparation”. The remaining media then underwent serial centrifugation at 2,000 g, 10,000 g, and 100,000 g for 30, 45, and 120 minutes respectively at 4 °C to isolate an EV-pellet enriched with small vesicles, while the supernatant was collected and defined as “EV-depleted media” preparation. The EV pellet was re-suspended in 2–3 mL of PBS and further purified by passing through the qEV size-exclusion purification column (Izon, Cambridge, USA) according to manufacturer’s instructions to remove soluble protein contaminants. These purified EVs were then centrifuged again at 100,000 g for 2 hrs and the pellet was re-suspended in different buffers depending on the experiment. For functional assays, the pellet was re-suspended in the original volume of placenta explant media used for culture (“EV-enriched media”) to allow direct comparison of the relative roles of EVs and soluble components on the effects elicited by total conditioned media. 10-fold EV-enriched media from sPE and 1^st^ trimester-derived tissues was also prepared. Due to instrument unavailability, we did not quantify EVs density however total protein concentrations in EV-enriched preparations, across gestation and in sPE, were similar (range 94–155 mg/ml). For Western blot analyses, EVs were directly lysed with lysis buffer and total protein content was analyzed by the BCA assay (Thermo). The pellet following the 10,000 g spin that is enriched in larger micro-vesicles and apoptotic bodies was also collected for ELISA analyses.

### Transmission Electron Microscopy (TEM)

TEM on freshly isolated EV preparations, re-suspended in PBS, were performed at the Advanced Bioimaging Centre at Sinai Health Center, Toronto, Canada by Mr. Doug Holmyard.

### Leukocyte Adhesion Assay

Fifty thousand immortalized human aortic endothelial cells (TeloHAEC from ATCC) were seeded onto 96-well plate and grown to form a confluent monolayer (24 hrs). Cells were then stimulated with 50% treatment media or positive control (50 ng/mL of TNFα) diluted in growth media for 6 hours. THP-1 monocytes (ATCC) were washed with PBS, and then labelled with 1 µM calcein-AM (Thermo Fisher) in PBS for 20 minutes at room temperature. Labelled monocyte cells were washed once with PBS and then re-suspended in complete endothelial growth media (EBM + EGM-2MV bullet kit, (Cedarlane, Burlington, ON, Canada)). Following stimulation, endothelial cells were washed, and then 50,000 THP-1 cells/well were overlaid on top of the endothelial cells and incubated for 45 minutes. Following the incubation at 37 °C, non-adherent cells were washed 3 times with PBS prior to fluorescent read out at 480 nm excitation and 520 nm emission using the Infinite M200 plate reader (Tecan, Switzerland). Results were normalized to cells treated with 2X complete endothelial media with 50% placental explant media (See angiogenesis section for details on media preparation).

### Western blotting

Equipment and reagents used for Western blots were from Bio-Rad, Mississauga, ON, Canada unless otherwise stated. Thirteen µg of purified EV total protein (lysed in RIPA or NP40 buffer), and 4X Laemmli sample buffer loading dye with or without 10% β-mercaptoethanol, respectively, were electrophoresed in 1x TG-SDS Buffer on TGX Stain-Free™ Precast gels. Following electrophoresis, UV activation of the gel was performed and the gels were imaged using the manufacturer’s protocol. Proteins were transferred onto low-fluorescence PVDF membrane using the Trans-Blot Turbo Transfer System. Total protein intensity was imaged using ChemiDoc™ MP system according to manufacturer’s protocol. Membranes were blocked in 5% milk/TBS-T for 1 hour at room temperature. All primary antibodies were incubated overnight at 4 °C in 5% milk/TBS-T. Following washes with TBS-T, membranes were incubated for 1 hour at RT in the appropriate secondary antibodies diluted to 1:3,000 in 5% milk-TBST. Membranes were developed using Western Lightning *Plus*-ECL (Thermo Scientific) using the ChemiDoc™ MP system. Band intensities within the linear range were quantified using Image Lab software. Proteins of interest were normalized to total protein intensity in place of housekeeping genes.

### Angiogenesis

Ten thousand human umbilical vein endothelial cells (HUVECs) in 100 µl of media were cultured on 50 µl of growth factor-reduced Matrigel (Corning, Tewksbury, MA, USA) for 20 hrs with the following treatments; (a) positive control (complete endothelial growth media – EBM + EGM-2MV bullet kit, (Cedarlane)), (b) 50% untreated placental explant media, (c) 50% total-conditioned media, (d) 50% EV-depleted media and (e) 50% EV-enriched media. The untreated placental explant media control and conditioned treatment media (b–e) were prepared by diluting the media with 2X complete HUVEC media at 1:1 ratio. This dilution ensured optimal growth factor concentration for the HUVECs while obtaining 50% (by volume) of conditioned media. Positive control (complete HUVEC media) and diluted placental explant media with 2X complete HUVEC media (1:1 ratio) produced equal results in all of the functional assays tested, therefore in all subsequent experiments the diluted placental-2X HUVEC media was used as a positive control. Images were taken at 4X magnification after 20 hrs of incubation using MicroPublisher 5.0 RTV camera (QImaging, Surrey, BC, Canada) mounted to DMIL LED inverted light microscope (Leica, Concord, ON, Canada) and analyzed for total tubule length using ImageJ Software.

### Fluorescent labeling of EV

Purified EVs were labeled using PKH67 fluorescent cell linker kit according to the manufacturer’s specifications (Sigma, Oakville, ON, Canada). EVs re-suspended in the provided diluent were added to 2 µl of dye in 500 µl of diluent and incubated at room temperature for 5 minutes. Labelled EVs were then spun down at 100,000 g for 1 hr, washed in PBS, centrifuged again and then re-suspended in 150 µl of PBS.

HUVEC or MCF7 cells were grown overnight in 16 well tissue culture chamber slide (Thermo Fisher). The next day, media was replaced with 100 µl of fresh complete media and 10 µl/well of fluorescently labelled EVs. The cells with labelled EVs were placed in an incubator for 4 hrs. Cells were then washed with PBS to remove no-adherent EVs and fixed for 5 min with ice cold Methanol:Acetone (1:1) mixture. After fixation cells were washed with PBS and DAPI staining was performed prior to cover-slipping. The slides were imaged using WaveFX Spinning Disc Confocal System by Quorum (Guelph, Ontario, Canada) with optimized Yokogawa CSU X1, Hamamatsu EM-CCD digital camera Image EM (C9100-13), and Leica DMI6000B inverted research grade motorized microscope run by Volocity 6.3.0 Acquisition software (Improvision/Perkin Elmer, Massachusetts, USA). Mean fluorescent intensity was measured using Volocity 6.3.0 software.

### Gene Expression Studies and qRT-PCR

Twenty five thousand HUVEC cells were seeded in 96 well plates overnight. The following day the cells were treated for 24 hrs with (a) control (complete endothelial growth media), (b) total placental explant media, (c) total-conditioned media, (d) EV-depleted media and (e) EV-enriched media. The media was prepared by diluting it with 2X complete media (please see angiogenesis section for details). Following treatment, RNA was extracted from the cells using RNeasy Plus Mini Kit (Qiagen, Toronto, ON, Canada) according to the manufacturer’s recommendations. Five hundred nanograms of each sample was reversed transcribed to cDNA using iScript^TM^ Reverse Transcription Supermix (Bio-Rad) according to the manufacturer’s instructions. Gene expression was measured using quantitative real time-PCR and run on the CFX384 Real-Time PCR Detection System (Bio-Rad) with LuminoCT® SYBR® Green qPCR ReadyMix^TM^ (Sigma). Gene expression was normalized to the geometric mean of three housekeeping genes (TBP, YWHAZ, TOP1). Gene of interest expression in each treatment was expressed as fold change relative to its respective control. Primer sequences are shown in Supplementary Table 2.

### Enzyme-linked Immunosorbent Assays (ELISA)

Angiogenic protein concentrations were quantified in total-conditioned, EV-depleted, EV-enriched and macro vesicle/apoptotic body enriched media as well as in RIPA lysed EV pellets using ELISAs from R&D Systems (Burlington, ON, Canada). The following kits: Quantikine Human Endoglin/CD105 (cat #DNDG00), Quantikine Human PlGF (cat #DPG00) and Quantikine Human VEGF R1/Flt-1 (cat #DVR100B) were used according to manufacturer’s specifications. Plates were read using Infinite M200 plate reader (Tecan).

### Proliferation and Cytotoxicity assays

One hundred fifty thousand HUVEC cells were seeded in 24 well plates overnight. The following day the cells were treated for 24 hrs with (a) control (complete endothelial growth media), (b) total placental explant media, (c) total-conditioned media, (d) EV-depleted media and (e) EV-enriched media. The media was prepared by diluting it with 2X complete media (please see angiogenesis section for details).

Proliferation and cytotoxicity assays were performed on treated HUVEC cells utilizing the CellTiter 96® AQ_ueous_ One Solution Cell Proliferation Assay (Promega, Madison, USA) and the *In Vitro* Sulforhodamine B Toxicology Assay Kit (Sigma-Aldrich) according to the provided specifications.

### Statistics

All experiments were carried out in at least biological quadruplicates. All the values are shown as mean ± the standard error. Western blot data and EV entry intensity scores were analyzed using t-test with Mann-Whitney correction. Angiogenesis and leukocyte adhesion assays were analyzed using column statistics – one sample t-test. Gene expression and ELISA data was analyzed using one-way ANOVA with Bonferroni’s multiple comparisons test and between group comparisons was performed using t-test. sFlt-1:PlGF ratios were analyzed using two-way ANOVA with Bonferroni *post hoc* analysis.

### Data availability

Original data is available upon request.

## Electronic supplementary material

Supplementary Information
