# Supplementary material for: Endothelial Dysfunction in Severe Preeclampsia is Mediated by Soluble Factors, Rather than Extracellular Vesicles
Source: Sci Rep. 2017 Jul 19;7:5887. doi: 10.1038/s41598-017-06178-z (PMC5517616; doi:10.1038/s41598-017-06178-z)
Supplement: Supplementary file 1 — Supplementary Information [file 41598_2017_6178_MOESM1_ESM.pdf]

ENDOTHELIAL DYSFUNCTION IN SEVERE PREECLAMPSIA IS MEDIATED BY SOLUBLE FACTORS, RATHER THAN EXTRACELLULAR VESICLES

Michelle O'Brien, BSc (1)
Dora Baczyk, MSc (1)
John C. Kingdom MD (1,2,*)

From the Research Centre for Women's and Infant's Health, Lunenfeld Tanenbaum Research Institute (1) and the Department of Obstetrics & Gynecology, Maternal-Fetal Medicine Division (2), Mount Sinai Hospital, University of Toronto, Ontario, Canada.

Supplementary Figure 1

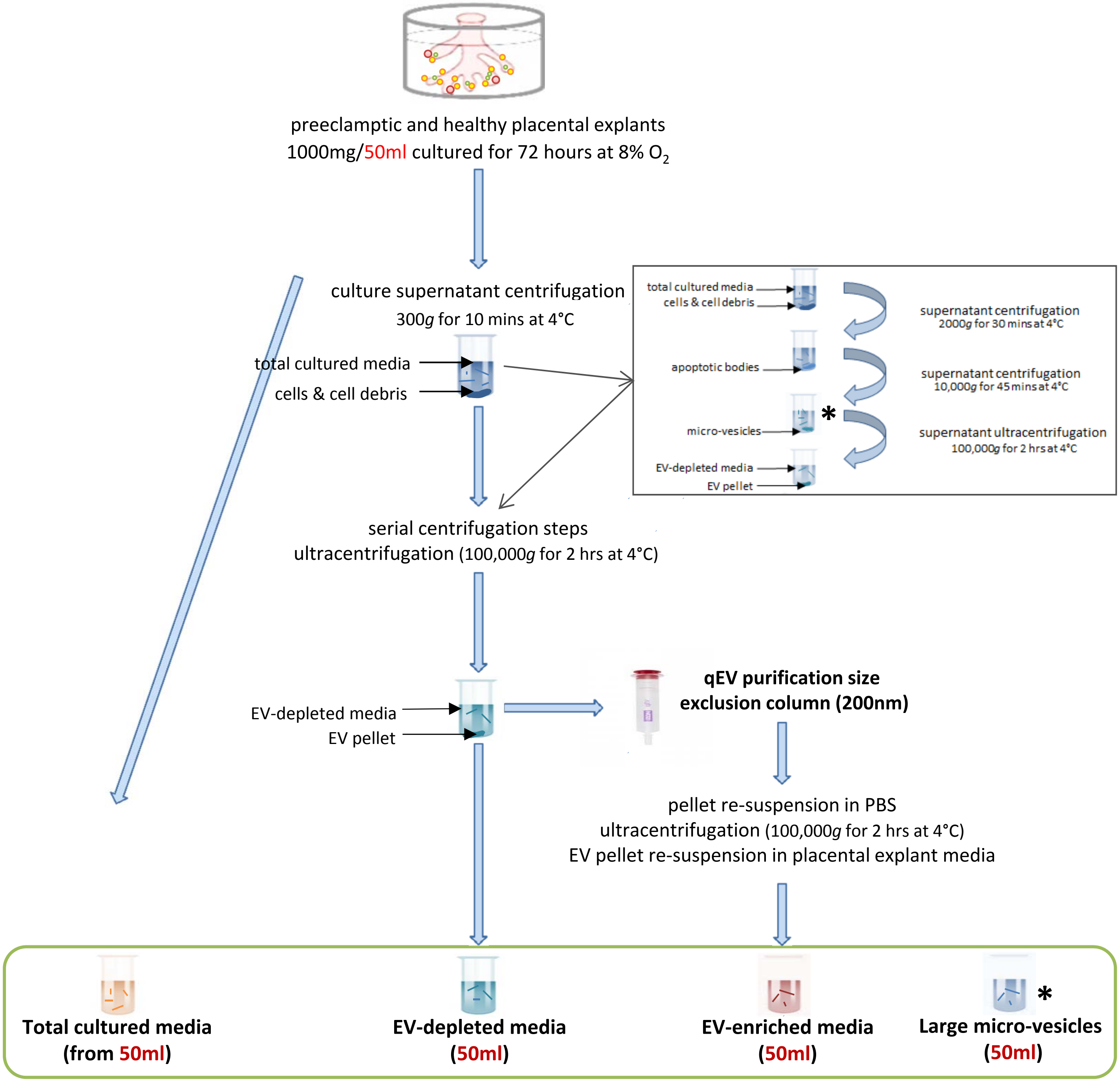

Supplementary Figure 2

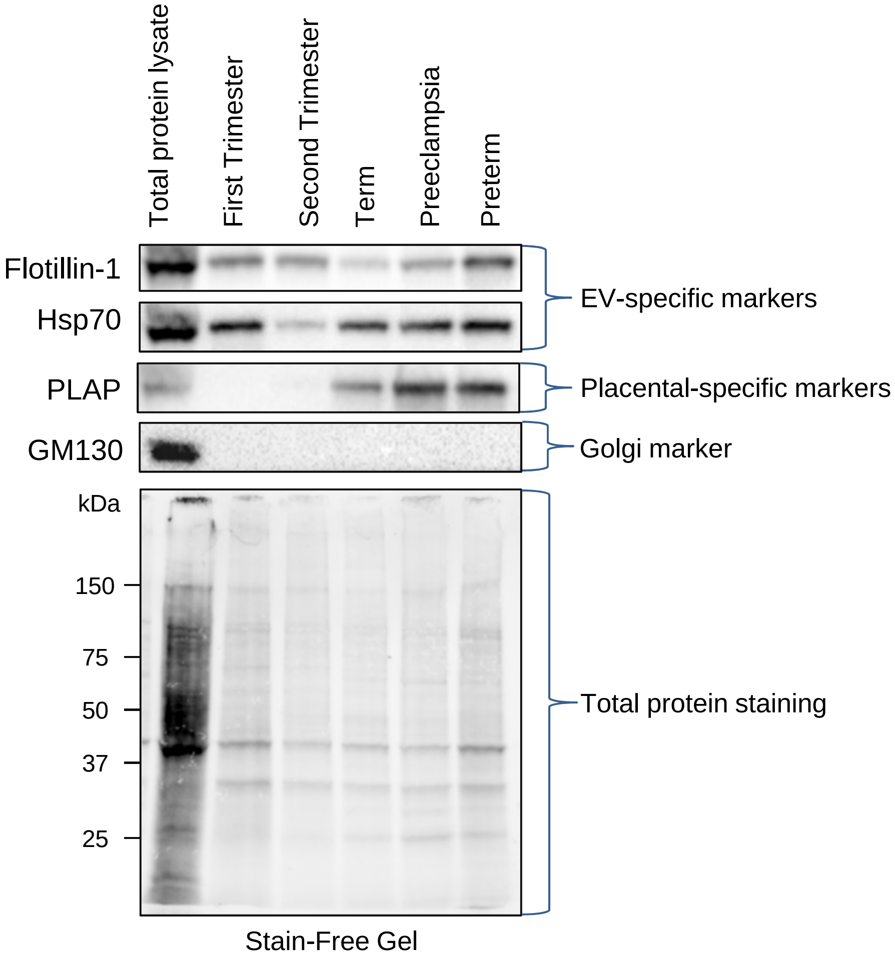

Supplementary Figure 3

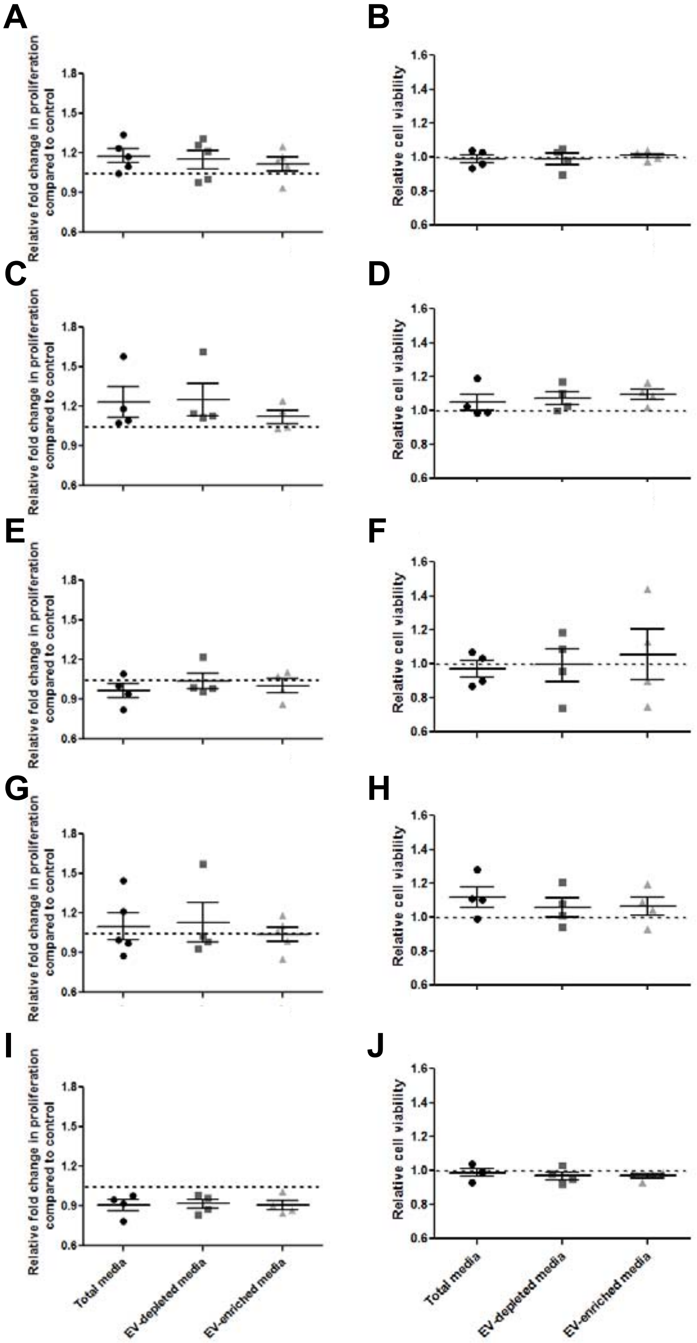

Supplementary Table 1: Patient information

		Preterm	Term	sPE	1st Trimester	2nd Trimester
	**Number**	4	16	11	14	10
	**Gestational age (weeks)**	29.0 ± 2.8	38.9 ± 0.8	29.5 ± 2.1	8.76±1.6	18.0±1.8
	**Maternal Age (years)**	31.0 ± 2.9	34.9 ± 4.1	34.6 ± 5.5		
	**Gravida**	1.8 ± 1.0	2.2 ± 1.0	2.0 ± 1.8		
	**Para**	0.3 ± 0.5	0.9 ± 0.9	0.8 ± 1.5		
**Delivery Method**	**Vaginal**	2	0	0		
**Ethnicity**	**Black**	0	1	1		
	**Caucasian**	3	12	5		
	**Asian**	0	1	1		
	**East Indian**	1	2	3		
	**Middle Eastern**	0	0	1		
	**N/A**	0	0	0		
**BP ≥140/90mmHg**	**No**	4	14	0		
	**Yes**	0	0	11		
	**N/A**	0	2	0		
**Abnormal Umbilical cord Doppler (AEDV,REDFV)**	**No**	4	0	3		
**Abnormal Umbilical cord Doppler (AREDFV)**	**Yes**	0	0	8		
	**N/A**	0	15	0		
**Sex**	**Male**	3	5	6		
	**N/A**	0	15	0		
	**Female**<br>**N/A**	1<br>0	11<br>0	5<br>0		
	**Birth weight (g)**	1348 ± 498	3468 ± 562	997 ± 291		
	**N/A**	0	0	0		
**Placenta**	**weight (g)**	380 ± 124	N/A	256 ± 74		

N/A=not available

AREDFV=absent/reversed end-diastolic flow velocity

Supplementary Table 2: Primer sequences

Primer Name		Primer Sequence
TBP	forward	TGC ACA GGA GCC AAG AGT GAA
	reverse	CAC ATC ACA GC CCC CAC CA
TOPO1	forward	GAT GAA CCT GAA GAT GAT GGC
	reverse	TCA GCA TCA TCC TCA TCT CG
YWHAZ	forward	ACT TTT GGT ACA TTG TGG CTT CAA
	reverse	CCG CCA GGA CAA ACC AGT AT
Endoglin	forward	CATGCAGATCTGGACCACTG
	reverse	GCCACAATGCTGGCATTGAG
eNOS	forward	GAA GCT GCA GGT GTT CGA TG
	reverse	CCG GTT GGT GGC ATA CTT GA
Endothelin1	forward	GAA CTC AGG GCT GAA GAC AT
	reverse	GGT GTT CCT CTG AAC TTC TTC TG
SOD1	forward	GAC TGA CTG AAG GCC TGC AT
	reverse	CAC TGG TAC AGC CTG CTG TA
TGFß	forward	TGC TGC AGG CCT TCG ATG T
	reverse	GAC TCC CTC TGG CAA TTG C
PlGF	forward	CAC GTG GGC CCT GCT TGC TG
	reverse	ACA CTT CTT GGA AGG GTA CCA
sFLT	forward	TGG ACT GAC AGC AAA CCC AA
	reverse	CAG AAA CTG GGG CTG CTG AC
HO1	forward	CCG CTT CAA GCT GGT GAT GCC
	reverse	CCT GCT CCA GGG CATG CCT TG
HO2	forward	CGG CTC CAC ACT GGG CAG AG
	reverse	GCC CTC CAG GGC ACC TTT GTC
ICAM	forward	GAC CGC AGA GGA CGA GGG CA
	reverse	TTG GGC GCC GGA AAG CTG TAG
MCP1	forward	TTC ATT CCC CAA GGG CTC GCT CA
	reverse	AGC ACA GAT CTC CTT GGC CAC AA

Supplementary Figure 1. Schematic depiction of serial centrifugation and purification steps employed to fractionate: total, EV-depleted, EV-enriched media and micro-vesicle enriched fractions.

Supplementary Figure 2. Western blot images of EV preparations from healthy and PE placentas. Total protein staining was used for normalization analysis. Total protein lysate = pool of first, second and third trimester as well as preterm and PE explants.

Supplementary Figure 3. The effects of total, EV-depleted and EV-enriched media on the proliferation and toxicity of HUVECs was assessed. Proliferation of HUVES (left column) was induced by incubation with media from (A) first trimester, but not altered by incubation with (C) second trimester, (E) preterm, (G) term or (I) PE placentas. Treatment of HUVECs with (B) first trimester, (D) second trimester, (F) preterm, (H) term and (J) PE media did not induce toxicity.
